# Cerebro-Spinal Meningitis

**Published:** 1866-12

**Authors:** F. R. Payne

**Affiliations:** Marshall, Ill.


### CHICAGO MEDICAL EXAMINER.

N. S. DAVIS, M.D., EDITOR.

VOL. VII. DECEMBER, 1866. NO. 12.

### Original Contributions.

### ARTICLE XLII.

### CEREBRO-SPINAL MENINGITIS.

BY F. R. PAYNE, M.D., Marshall, Ill.

Read to the Illinois State Medical Society, June, 1866.

It would afford me great pleasure to meet with you on the 5th of June, 1866, but circumstances render it impossible for me to do so. We have, in this county, had three years of sad experience of the *epidemic* prevalence of "spotted fever." During this time, we have learned some practical facts relating to this dreadful epidemic. We may have sporadic cases of this disease, and the results of our observations are, that they are generally curable. In the last 24 years, we have had three terrible epidemics in this county, and, in my opinion, they have all been, pathologically, identically the same, the only difference being in the premonitory symptoms. This year, we have carefully noted, at the bedside, the symptoms of *epidemic* cerebro-spinal meningitis, and in all cases where death does not result in a few hours we have two stages.

*First Stage.*—Congestion, not a well-marked chill, but tremor; surface cold, and, in nearly every case, pulse not perceptible at the wrist; the skin an ash or lead color; excruciating pain in head and back of neck; restlessness and vomiting, with a tremulous sensation in the epigastrium; the entire surface of the body very sensitive, but often we *early* have paralysis of one or more of the extremities. If reaction is not established in a few hours, we have loss of sight and hearing; stiffness of muscles of neck; local spasms, with a flexed rigidity of fingers and toes of one or both sides of the body. If these symptoms continue for six to twelve hours, we have cessation of respiration. In the *genuine epidemic*, if we are called promptly, and succeed in establishing reaction, we soon have a new train of symptoms, which we denominate

*Second Stage.*—We now have a quick, hard, small pulse; suffused eyes; the neck rigid, and more or less opisthotonos; great restlessness and jactitation; in many cases, petechiæ on limbs or body; bowels constipated, and in many cases impossible deglutition; and complete coma. The pupils of the eyes in this stage begin to contract, but if the patient lives three or four days, we have permanent dilatation. These symptoms are magnified by the upright position. In the first stage, the pupils will dilate and contract without an effort to produce this change; in the second stage, one or both pupils are permanently dilated and are not influenced by exposure to intense light.

This epidemic has prevailed in Clark County for three years, but in no locality longer than four to six months; and it is positively certain that the disease is migratory. The first cases were at York and Darwin, 10 and 20 miles south of this point. These cases occurred in 1863–64, and many died. In November, 1864, the terror of the disease was felt in this place, and none but an occasional *sporadic* case was known to exist in the localities of Darwin or York. In the spring of 1866, the epidemic reached the northern part of this county, some 10 and 15 miles north of town, and we had no cases in Marshall or vicinity. We have carefully noted the condition of the atmosphere in the localities where the disease prevails. Four or five days prior to the outbreak of the epidemic last spring, north of Marshall, we had damp, foggy weather. This was also true in this locality when the disease prevailed.

We have, in this malady, congestion and depression, indicating some toxic agent in the blood which seems to have an elective affinity for the brain and spinal marrow. The vitality of the blood is destroyed and rapidly disorganized, and the vessels of the brain and spinal marrow seem to lose their contractility. If this is true, we have a specific poison at work, and nothing but *elimination* or *neutralization* will stop the mischief.

In 1845–46, we had a terrible epidemic in this county, called "black tongue." This disease first made its appearance in the form of ordinary erysipelas, but in a few days extended through the mucous membranes to internal parts; then we had depression, congestion, delirium, and all the symptoms now found in spotted fever.

In 1858, a similar and fatal epidemic prevailed, which we denominated *erysipelas of the brain.* The symptoms in this disease were nearly the same as are now found in "spotted fever."

It is the opinion of JOSEPH KLAPP, M.D., and many other medical men, that the poison is the same as that of *erysipelas;* and the facts here presented surely favor that idea. Prof. N. S. DAVIS says:—"There is not only a close analogy, but a positive relation between erysipelas and epidemic cerebro-spinal meningitis, or spotted fever; the two diseases having often prevailed together in the same community, and in members of the same family." Prof. DAVIS is one of the ablest practical physicians in the North-west, and his observations are worthy of the highest consideration. We are, and may long remain. ignorant of the primary essential or specific cause of this disease, but, in our opinion, the pathological condition is abundantly obvious. Prof. N. S. DAVIS truly and forcibly says:—"the practitioner who stands by, watching his patient laboring under a severe attack of this disease, and sees the rapidity with which the nervous, vascular, and secretory functions all fail, as indicated by the muscular rigidity and delirium, passing into paralysis and coma; by the universal capillary congestion and frequent appearance of purple spots; by the suspension of important secretions; and by the rapid development of sero-purulent infiltrations and effusions wherever local hyperæmia or inflammation exists, will not fail to recognize a profound alteration of the relations of affinities between the ingredients of the blood and the organized structures of the body."

If our pathological position is true, all must admit the necessity of a prompt resort to some medicinal agent that will arrest this septic or degenerative tendency. If this is not speedily accomplished, death is positively certain. In the first stage, if we have an *epidemic case*, we must, in some way, procure reaction; if not, our patient dies in a few hours—they are blue, cold, pulseless, and die in from six to twelve hours. I know of no better remedies to meet this indication, than hot rocks, mustard to the spine, rub with pepper and whiskey, and use with caution brandy internally, and, at the same time, resort promptly to the use of antiseptics, the most reliable of which are the arsenites and permanganates. The most valuable are the arsenite of potassa and permanganate. It is also true, that in the treatment of erysipelas the profession is almost unanimously favorable to tinct. ferri chlor. Dr. JOSEPH KLAPP, in the *Medical & Surgical Reporter*, April 30th, 1864, states that he has successfully treated many cases of this disease by giving 20 drops of mur. tinct. ferri every two hours, until the cerebral symptoms began to give way, and then he added 1 gr. sulph. quinine, as a tonic, to each dose; generous diet, milk-punch, if necessary, and perfect rest of body and mind, requiring in all cases the recumbent position. Dr. KLAPP says the following liniment will relieve the distressed feeling at the back of the head:—

℞. Tinct. Aconite Rad.,------------------

Tinct. Opii, --------------------------āā f ℥ss

Sapon. Comp.,------------------------- f ℥iij

Ft. Liniment.

With this treatment, he says, "I have relieved some distressing cases."

The history of the epidemic in this county is, that when it first appeared in a locality it was much more fatal than it was after the disease had prevailed for some three or four weeks, and in some localities it has been more virulent than in others. It is also true, that it selects for its victims persons between the ages of 2 and 15 years. The territory in which the disease prevailed, did not, each year, comprise over one township. The march of this epidemic atmosphere has, thus far, been north and a little west. If its future progress is in accordance with that of the last three years, the disease will reach Grandview, Edgar Co., in the spring of 1867. When this dreaded malady first appears in a neighborhood in an *epidemic form*, three-fourths of all the *genuine* cases will die. In ten or twelve days after the breaking out of the disease, more than half the cases will be relieved with the treatment above indicated. The permanganate of potassa or the arsenite of potassa ought to be given from the inception of the disease, and in localities where intermittents prevail, we prefer the latter, that is Fowler's solution, 3 to 5 drops every four hours; we also use tinct. ferri chlor. in 10 to 20 drop doses.

Last spring, in one family, we had four well-marked cases. One, a stout, hearty man, died in eight hours after first symptoms made their appearance; two got well; the other, a young lady, lived eight weeks, with complete paralysis of the inferior extremities. More than half of this time her appetite was good; bowels regular; no fever; tongue clean, but the recuperative powers of the system failed to restore health, and she died. The pathology was well-marked, consisting in a highly pyæmic or septic condition of the blood, with visible perverted vital affinity.

We freely confess that our treatment in *epidemic* "spotted fever" is not satisfactory, but we hope the medical profession will soon present medicinal agents which will vitalize the blood and enable us to arrest that terrible septic and degenerative tendency.

My object in presenting this paper to your Society, is to elicit discussion, and thus secure to the profession all important pathological and practical facts which will in future enable us to better control this terrific malady.

